# Commentary: Can Blood Flow Restricted Exercise Cause Muscle Damage? Commentary on Blood Flow Restriction Exercise: Considerations of Methodology, Application, and Safety

**DOI:** 10.3389/fphys.2020.00243

**Published:** 2020-03-20

**Authors:** Mathias Wernbom, Brad J. Schoenfeld, Gøran Paulsen, Thomas Bjørnsen, Kristoffer T. Cumming, Per Aagaard, Brian C. Clark, Truls Raastad

**Affiliations:** ^1^Center for Health and Performance, Department of Food and Nutrition and Sport Science, University of Gothenburg, Gothenburg, Sweden; ^2^Department of Health and Rehabilitation, Institute of Neuroscience and Physiology, Sahlgrenska Academy, University of Gothenburg, Gothenburg, Sweden; ^3^Department of Health Sciences, CUNY Lehman College, Bronx, NY, United States; ^4^Department of Physical Performance, Norwegian School of Sport Sciences, Oslo, Norway; ^5^Department of Sport Science and Physical Education, Faculty of Health and Sport Sciences, University of Agder, Kristiansand, Norway; ^6^Department of Sports, Physical Education and Outdoor Studies, Faculty of Humanities, Sports and Educational Science, University of South-Eastern Norway, Notodden, Norway; ^7^Department of Sports Science and Clinical Biomechanics, University of Southern Denmark, Odense, Denmark; ^8^Ohio Musculoskeletal and Neurological Institute, Ohio University, Athens, OH, United States; ^9^Department of Biomedical Sciences, Ohio University, Athens, OH, United States

**Keywords:** occlusion, ischemia, hypoxia, muscle fiber degeneration, muscle hypertrophy, fatigue

Low-load exercise combined with blood flow restriction (BFR) is known to induce significant gains in muscle strength and size, and this mode of training is increasingly used in both healthy and clinical populations, as documented in the recent review of Patterson et al. (2019). However, since the first training studies on BFR exercise appeared about 20 years ago, there have been some concerns about its safety, in particular with regard to the potential risk for muscle damage (Wernbom et al., 2019). In a recent editorial, Wernbom et al. (2019) briefly discussed the accumulating evidence for muscle damage and rhabdomyolysis with very strenuous and unaccustomed BFR resistance exercise (BFR-RE). In contrast, Patterson et al. (2019) stated that “analysis of the incidence rate from the published literature suggests the risk remains very low (0.07–0.2%),” referring to the editorial of Thompson K. M. A. et al. (2018). Patterson et al. (2019) went on to conclude: “In summary, the available evidence suggests that the application of BFR does not appear to induce a muscle damage response to low-load resistance exercise using single exercise protocols of up to five sets to volitional failure.” In our view, these statements do not recognize the nuances and complexities of the topic, and we argue that the available evidence does suggest that BFR-RE may induce muscle damage under some circumstances (Wernbom et al., 2019). Given the obvious importance of the issue, in this commentary we will elaborate on the points discussed in the recent editorial of Wernbom et al. (2019).

## Can Blood Flow Restricted Resistance Exercise Induce Muscle Damage and Rhabdomyolysis?

Exertional rhabdomyolysis is a well-known complication of extreme physical exertion and exhaustive exercise (Knochel, 1990; Clarkson et al., 2006; Thompson T. L. et al., 2018). The term rhabdomyolysis defines an injury to skeletal muscle cells of such severity that their contents leak into the circulation (Knochel, 1990). Muscle proteins that leak into the circulation include myoglobin, creatine kinase (CK), lactate dehydrogenase (LDH), alanine aminotransferase (ALT), aspartate aminotransferase (AST), and aldolase (Knochel, 1990; Clarkson et al., 2006). A level of >10,000 U/L of CK, which is >50 times higher than the normal upper limit, is generally accepted to be diagnostic of rhabdomyolysis, and a CK value of >2,000 U/L is commonly used to diagnose myopathy (muscle disease) (Clarkson et al., 2006). It should be noted that lower thresholds of CK have also been used, for example 5–10 times the baseline value, or ~1,000–2,000 U/L (Thompson T. L. et al., 2018; Bäcker et al., 2019), and it was recently suggested by Fernandes and Davenport (2019) that a rise in CK to >5,000 U/L is sufficient for a diagnosis of exertional rhabdomyolysis.

As noted previously (Wernbom et al., 2019), there are now no less than four published case reports of individuals experiencing rhabdomyolysis after a single session of BFR-RE (Iversen and Røstad, 2010; Tabata et al., 2016; Clark and Manini, 2017; Krieger et al., 2018), all reporting CK in excess of 10,000 U/L. Furthermore, at least two acute training studies (Yasuda et al., 2015; Sieljacks et al., 2016) on BFR-RE have reported high post-exercise CK levels, with some individuals displaying peak CK values consistent with a rhabdomyolysis diagnosis.

**Figure 1:**
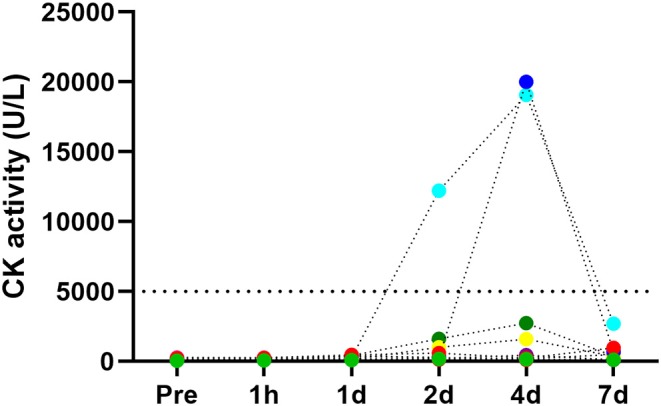
Responses in creatine kinase (CK) activity levels in serum before and 1 hour (1h), 1 day (1d), 2 days (2d), 4 days (4d), and 7 days (7d) after a damaging bout of low-load BFR-RE. Figure based on data from individuals in the study of Sieljacks et al. (2016). Note the differences between individuals, and also in the time-course of the responses in the two participants who were “high-responders” (CK > 19,000 U/L). The upper detection limit for the CK essay in this study was 20,000 U/l, and one of these two individuals may have exceeded this limit. Serum myoglobin showed similarly high increases (data not shown).

Sieljacks et al. (2016) investigated the responses in nine recreationally active but not resistance-trained men to a first-time BFR-RE session of five sets to failure of knee-extensions at 30% of one repetition maximum (1RM). The BFR cuff was 135 mm wide and inflated to a pressure of 100 mm Hg during exercise. With this cuff width, 100 mm Hg of pressure is typically ~50–60% of the complete arterial occlusion pressure (AOP) in the femoral artery in young male subjects during rest in a seated position (Wernbom et al., 2012). On average, a total of 59 repetitions were performed, with 24 repetitions in the first set and seven repetitions in the final set (Sieljacks et al., 2016). The mean peak CK value at 96 h after BFR-RE was 4,954 U/L. This high mean CK peak was mainly driven by the responses of two of the subjects who displayed peak CK values of >19,000 U/L, but two other subjects demonstrated peak levels of 2,747 and 1,585 U/L, respectively (Sieljacks et al., 2016). The individual responses are illustrated in Figure 1.

Similar to CK, myoglobin also displayed marked increases in the days following acute BFR-RE. In the same study, Sieljacks et al. (2016) also investigated the responses to 150 maximal eccentric knee extensions. The mean peak CK level (at 96 h post-exercise) in the eccentric exercise group was 2,936 U/L, i.e., less than reported in the BFR-RE group.

Yasuda et al. (2015) investigated the effects in 10 recreationally active men (three of them had light to moderate resistance-training experience) of a first-time BFR-RE session of four sets to failure of elbow flexions at 20% of 1RM for a mean total of 111 ± 36 repetitions. Their BFR-RE protocol involved a 30 mm wide Kaatsu-master cuff set at 160 mm of pressure. It deserves mention that despite the suprasystolic pressure, 160 mm Hg with the 30 mm Kaatsu-master cuff has been shown to induce only a moderate degree of BFR compared with the complete occlusion observed with the same cuff at 300 mm Hg (Yasuda et al., 2009). This combination of cuff width and pressure has been successfully employed to induce strength gains and muscle hypertrophy with longer-term BFR exercise with a fixed 30-15-15-15 repetition protocol (Yasuda et al., 2012). In their acute study on BFR-RE to contraction failure, Yasuda et al. (2015) reported mean peak CK values of 13,415 ± 7,267 U/L at 96 h post-exercise in the three subjects from which CK levels were analyzed. Closer examination reveals that the mean values and standard deviations reported by Yasuda et al. (2015) are only possible if all three subjects had several thousand U/L in CK, and two of them must have had CK values in excess of 10,000 U/L.

Interestingly, the values reported by Yasuda et al. (2015) are very similar to the 11,932 U/L in CK observed by Nosaka and Clarkson (1996) in subjects who had performed 24 maximal eccentric contractions. The pathophysiological significance of such high CK values is underscored by the observation that degenerating and necrotic muscle fibers are a relatively common finding in the muscles of subjects displaying several thousand U/L of CK after muscle damage induced by voluntary eccentric exercise (Jones et al., 1986; Round et al., 1987; Paulsen et al., 2010a,b), electrically stimulated eccentric contractions (Mackey et al., 2016), and voluntary eccentric contractions with superimposed electrical stimulation (Child et al., 1999). Accordingly, it has been suggested that delayed increases in CK of this magnitude likely reflect muscle fiber necrosis (Paulsen et al., 2010a). Along the same lines, Foley et al. (1999) proposed that destruction of a vulnerable pool of muscle fibers explained the observed 7–10% decreases in elbow flexor muscle volume at 2–8 weeks after a damaging eccentric exercise bout, which resulted in peak CK levels of 21,000 U/L. In this context, it deserves mention that peak CK levels typically precede the time point of peak numbers of necrotic and infiltrated muscle fibers (Jones et al., 1986; Round et al., 1987; Child et al., 1999).

Further support for possible muscle damaging-effects of exhaustive BFR-RE comes from a recent training study of Bjørnsen et al. (2019) on 13 subjects (nine men and four women) who were recreationally active and did not perform regular strength training. A mean CK of 1,224 ± 968 U/L and corresponding increases in serum myoglobin were observed after five training sessions in 4 days. One participant dropped out early because of severe muscle soreness and pronounced weakness in the quadriceps, which worsened after the fourth BFR-RE session to the extent that he could not continue training and had to walk with crutches for 2 days. His CK levels were 2,389 and 4,188 U/L on the third and the fourth day, respectively, vs. 194 U/L at baseline. Unfortunately, no blood samples were available from this individual at 4–6 days after the first session, when CK often peaks after severe muscle-damaging exercise (Jones et al., 1986; Child et al., 1999; Clarkson and Hubal, 2002; Paulsen et al., 2010b), but based on the extreme symptoms and the marked and apparently rising elevations in CK, it is reasonable to conclude that he developed rhabdomyolysis. Moreover, three of the 13 participants who completed the training study displayed CK values in the range between 1,800 and 2,550 U/L on the morning of the fifth day of training.

Collectively, the results from these studies along with the available case reports strongly support that BFR-RE can induce significant muscle damage and sometimes even rhabdomyolysis in otherwise healthy subjects, although this is likely dependent on factors, such as the training status of the individual as well as the degree of exertion and fatigue, as further discussed below. For a brief discussion on the possible mechanisms of muscle damage with excessive BFR-RE, as well as other signs of muscle damage that have been reported in the literature, we refer to the recent editorial of Wernbom et al. (2019).

## CK as a Marker for Muscle Damage and Necrosis

We recognize that CK is an indirect marker of muscle damage, and that as such it has obvious limitations and warrants caution in the interpretations. For example, CK levels are influenced not only by the time course of the processes that result in the release of CK from the affected muscle fibers and the severity of the damage, but also by the clearance of CK from the circulation (Clarkson and Hubal, 2002). Nevertheless, the rather consistent connection between rhabdomyolytic/near-rhabdomyolytic CK levels and degenerative muscle fiber changes observed in the studies on acute damaging eccentric exercise (discussed in the previous section) is further supported by classic studies which reported degenerating and necrotic muscle fibers in military trainees suffering from acute exercise-induced rhabdomyolysis (Greenberg and Arneson, 1967; Geller, 1973).

Finally, it is of note that in neuromuscular diseases, marked elevations of CK (sometimes more than 50- to 100-fold above normal) are seen primarily in myopathies in which there is a destruction of muscle fibers, such as the Duchenne and Becker muscular dystrophies, polymyositis, malignant hyperthermia, Miyoishi distal myopathy and necrotizing myopathy (Amato and Greenberg, 2013; Ansari and Katirji, 2014). Conversely, patients with myopathies in which the sarcolemma is intact often have a normal CK (Ansari and Katirji, 2014). Taken together, these observations strongly suggest that necrosis is a plausible cause for the high elevations in CK seen in rhabdomyolysis.

We acknowledge that this does not rule out the possibility of contributions from non-lethal cell changes (e.g., transient increases in membrane permeability, shedding of membrane blebs) to the overall increases in CK and in other muscle proteins in the blood. Even so, whether such mechanisms could theoretically cause rhabdomyolysis-like elevations in muscle proteins in the blood is unclear, and these would likely in any case be on the muscle injury continuum. However, this also warrants attention to an important point raised by Ansari and Katirji (2014) among others: normal or only mildly elevated serum CK levels do not necessarily exclude a myopathy. By extension, mild or no changes in CK do not exclude the possibility of detrimental changes with excessive BFR-RE, for example muscle fiber atrophy.

## Blood Flow Restricted Exercise: a Case for a Training-Overtraining-Muscle Damage Continuum

Based on the results of Sieljacks et al. (2016) and Yasuda et al. (2015), the incidence rate of rhabdomyolysis after acute BFR-RE would be as high as 22 and 67%, respectively, and the rate of exercise-induced myopathy would be 33 and 100%. Furthermore, the data from the training study of Bjørnsen et al. (2019) suggests that near-myopathic and myopathic CK levels occurred in 29% of the participants during the first week of training. These figures are in sharp contrast to the 0.07–0.2% incidence rates of rhabdomyolysis suggested by Thompson K. M. A. et al. (2018) and cited by Patterson et al. (2019).

However, our intent is not to suggest that such high incidence rates as 22–67% apply to BFR exercise in general. Specifically, we argue that, much like eccentric exercise, excessive exhaustive BFR-RE exercise can induce marked delayed elevations in CK and myoglobin consistent with the occurrence of exercise-induced muscle damage, and in some cases rhabdomyolysis, in healthy subjects unaccustomed to this type of training. Conversely, it seems reasonable to suggest that BFR-RE protocols that evoke only mild to moderate degrees of fatigue (i.e., moderate acute decreases in myocellular phosphocreatine and adenosine triphosphate stores, and in force capability) and/or involve modest volumes and durations of work are much less prone to induce signs and symptoms of muscle damage (Wernbom et al., 2019).

For example, Shiromaru et al. (2019) found no significant increases in muscle signal intensity on magnetic resonance imaging (MRI) scans obtained after 3 weeks of low-volume BFR-RE training with four sessions per week in young healthy but untrained men. The BFR-RE consisted of three sets of 15 repetitions of unilateral knee extensions at 30% of 1RM, with a BFR pressure of 80% of resting arterial occlusion pressure and 60 s inter-set rest periods. In contrast, Shiromaru et al. (2019) reported that the heavy resistance training for the other leg with three sets at 10 repetitions at 80% of 1RM for two sessions per week resulted in increases in MRI signal intensity at 3 weeks. Because an increase in signal intensity on MRI images is thought to reflect increases in water, the prolonged changes (several days) after damaging exercise are considered to indicate edema in the exercise-damaged muscle (Clarkson and Hubal, 2002). The recent finding of Sieljacks et al. (2019) of significantly less delayed-onset muscle soreness (DOMS) after four sets of submaximal effort (peak ratings of ~14–15 on a 6–20 Borg RPE scale) than four sets to failure of BFR-RE is also consistent with the notion of a training-overtraining-damage continuum in BFR-RE. It should be noted though that how DOMS relates to other markers of muscle damage after BFR-RE is at present unclear.

Nielsen et al. (2012) reported impressive increases in satellite cell numbers, muscle fiber areas and the number of myonuclei already after 7 BFR-RE sessions in 1 week, which however all showed no further increases with subsequent training weeks. The protocol was four sets of unilateral knee extensions to voluntary failure at 20% of 1RM with 30 s of rest between sets, at a pressure of 100 mm Hg with a 14 cm wide cuff. The subjects were young healthy males who did not perform any structured training regimes. Bjørnsen et al. (2019) attempted to improve upon the results of Nielsen et al. (2012), using a very similar BFR-RE protocol (four sets to failure at 20% of 1RM), including the same pressures and cuff model. Instead of fiber hypertrophy, Bjørnsen et al. (2019) found a temporary muscle fiber atrophy (especially in type II fibers) during and after the first week, along with more gradual increases in the number of satellite cells and myonuclei. The fiber atrophy had reversed at 3 days after the second training week and was followed by hypertrophy (19 and 11% for type I and type II fibers, respectively) at 10 days of detraining after the second training week, and the subjects appeared to peak in strength after 21 days of detraining (Bjørnsen et al., 2019).

**Figure 2:**
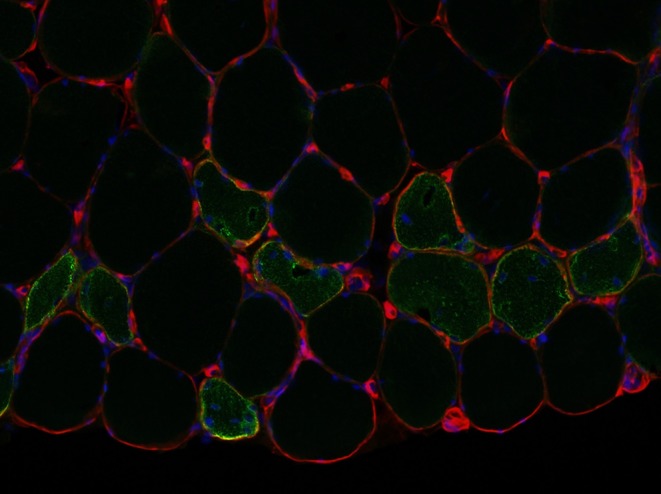
Small moderately strongly to strongly NCAM-positive muscle fibers from a subject in the study of Bjørnsen et al. (2019). The biopsy was taken 3 days after the last training week. Note central/non-peripheral myonuclei in several of the NCAM-positive fibers. The section was re-photographed (due to loss of the original pictures) after several years in the freezer, and the positive staining would likely have been even stronger if the section was new. Red = laminin, green = NCAM, and blue = DAPI. Picture cropped from 10× original. Picture courtesy of Mathias Wernbom.

Furthermore, whereas Nielsen et al. (2012, 2017b) found no apparently necrotic or regenerating muscle fibers, Bjørnsen et al. (2019) reported that a few subjects displayed small fibers which were strongly positive for the neural cell adhesion molecule (NCAM). NCAM-positive fibers are frequently encountered in biopsies from both muscular dystrophies and inflammatory myopathies in regenerating and denervated fibers, while necrotic fibers do not appear to express NCAM (Figarella-Branger et al., 1990; Winter and Bornemann, 1999). However, atrophic fibers in dermatomyositis patients and fibers with rimmed vacuoles in inclusion-body myositis also strongly express NCAM (Figarella-Branger et al., 1990). Two out of the 13 subjects in the study of Bjørnsen et al. (2019) displayed small strongly NCAM-positive fibers during the training period and such fibers were not seen in the pre-training biopsies (Wernbom, unpublished observations). The significance of these NCAM-positive fibers awaits further investigation, and examples of these are shown in Figure 2.

Importantly, the total number of repetitions per session was considerably higher in the study of Bjørnsen et al. (2019) compared to Nielsen et al. (2012, 2017a), particularly during the first three sessions (~80 vs. ~45 repetitions), and the level of exertion was likely also greater (for discussion, see Bjørnsen et al., 2019). Finally, it is noteworthy that Nielsen et al. (2017a) reported essentially no DOMS during the entire training period, whereas Bjørnsen et al. (2019) reported significant DOMS during the first training week, peaking at 39 mm on a 100 mm visual analog scale. As discussed earlier, CK levels also increased during the first 4 days. The muscle fiber atrophy, the elevated CK levels and the decreased strength during the first training week, which were followed by delayed hypertrophy and strength gains with detraining, are consistent with a temporary overtraining effect. This could conceivably explain much of the discrepancies in the results and time-courses between the studies of Nielsen et al. (2012) and Bjørnsen et al. (2019).

Collectively, these findings suggest that with high-frequency low-load BFR-RE, there is a limit in the volume and/or the level of exertion and overall stress imposed on the exercising muscles beyond which counterproductive effects on neuromuscular adaptations start to appear. It may also be speculated that this applies to a certain (albeit lesser) extent with BFR-RE at more normal training frequencies (e.g., 2–3 sessions per week). This could help explain why low-load BFR-RE to concentric contraction failure did not result in greater increases in muscle strength and size than BFR-RE with submaximal exertion after 8 weeks of thrice-weekly training (Sieljacks et al., 2019). In addition, high-volumes of low-load BFR-RE could result in more of a local endurance training stimulus, which may attenuate the hypertrophic responses (discussed in Wernbom and Aagaard, 2020 and Sieljacks et al., 2019).

## The Repeated Bout Effect in BFR-RE

We first proposed the existence of a “repeated bout effect” (i.e., less signs of muscle damage after a second training session) in BFR-RE 12 years ago (Wernbom et al., 2008), based on observations from our experiments on acute bouts of BFR-RE. In confirmation of this effect, Sieljacks et al. (2016) reported lower increases in CK and DOMS and less decrements in muscle strength after a second BFR-RE bout when the second session was performed 14 days after the first. Other studies have also reported results consistent with a repeated-bout effect in short-term BFR-RE (e.g., Farup et al., 2015; Bjørnsen et al., 2019; Sieljacks et al., 2019). An attenuation of the damage and stress responses with repeated sessions has implications for the prescription and safety of BFR-RE, not least with reference to the progression of important variables, such as the level of exertion, volume and frequency of training. However, this does not exclude that suboptimal and counterproductive effects could still occur with very strenuous BFR-RE, especially with high training frequencies and volumes.

## Can Ischemic Preconditioning Prevent Damaging Effects of Excessive BFR-RE on Muscle Fibers and the Endothelium?

It was recently demonstrated that ischemic preconditioning (IPC), i.e., repeated cycles of short periods of ischemia followed by reperfusion, can markedly blunt the delayed elevations in CK and DOMS and attenuate the decrements in muscle contractile twitch responses after high-force eccentric exercise (Franz et al., 2018). The IPC intervention in the study of Franz et al. (2018) was completed 5 min before the eccentric exercise bout.

In an interesting parallel, it has been shown that IPC can largely prevent signs of ischemia-reperfusion damage to muscle tissue resulting from exhaustive isometric ischemic exercise (Rongen et al., 2005). The ischemic exercise model of Rongen et al. involves 5 s contractions and 5 s relaxations repeated until exhaustion at 50% of MVC, with ischemia (200 mm Hg) maintained for 10 min regardless of the exercise duration. This model has repeatedly been demonstrated to injure the working muscles as judged by the increased uptake of technetium-99m–labeled Annexin A5 on Annexin A5 scintigraphy pictures (Rongen et al., 2005; Riksen et al., 2006; Draisma et al., 2009). Annexin A5 is an endogenous protein that binds with high affinity to negatively charged phosphatidylserine (PS). PS is located almost exclusively on the inner leaflet of the lipid bilayer of the normal cell membrane, but early in the process of apoptosis, the asymmetric distribution of PS is lost, and PS is exposed on the outer surface of the cell, thus providing binding sites for extracellular Annexin A5 (Rongen et al., 2005).

The cellular damage associated with the ischemic exercise protocol of Rongen et al. appears to be of a reversible nature and has been described as mild (Draisma et al., 2009), and it is not clear whether the injury occurs in the endothelium of blood vessels or in the muscle fibers, or both. However, endothelial and muscle function were not monitored in the days following the ischemic exercise bout in these studies, and delayed negative effects thus cannot be excluded, given that the highest CK levels seem to appear around 72–96 h after damaging bouts of BFR-RE (Yasuda et al., 2015; Sieljacks et al., 2016).

Damage to the blood vessels could in turn lead to delayed muscle fiber damage via local hypoxia, similar to the scenario in the “vascular hypothesis” proposed by Grundtman and Lundberg (2009) for the pathogenesis of idiopathic inflammatory myopathies (IIMs). The vascular hypothesis has some support in the observations of microvessel disturbances in dermatomyositis and polymyositis, two of the major subtypes of IIMs (Grundtman and Lundberg, 2009), and in the low tissue oxygen pressures that have been directly measured in the lower limb muscles of polymyositis patients (Kunze, 1970; Niinikoski et al., 1986).

The obvious similarities between low-load BFR-RE, in which pressures of up to 80% of AOP have been advocated (Patterson et al., 2019), and the ischemic exercise model of Rongen and colleagues suggest that their findings may be highly relevant also to BFR-RE. Indeed, one short-term BFR-RE study (Credeur et al., 2010) reported decreased flow-mediated dilation (FMD), suggesting impaired endothelial function. In contrast, other BFR-RE studies have shown improved FMD after periods of training (Evans et al., 2010, Patterson and Ferguson, 2010; Hunt et al., 2013). These discrepant results may depend on differences in the overall stress of the training sessions, and point to the urgent need for a better understanding of both the negative and positive effects of BFR-RE on endothelial function.

It also remains to be shown whether IPC before a very strenuous acute bout of BFR-RE can attenuate elevations in blood levels of CK and myoglobin as well as other markers and symptoms of muscle damage.

## The Potential Risk for Excessive Muscle Stress and Damage With Strenuous BFR-RE—Implications for Exercise Prescription and Research

In this Commentary, we have discussed evidence which supports that low-load BFR-RE can induce both beneficial and detrimental effects in skeletal muscle, depending on the circumstances. It is noteworthy that the training protocols employed in the studies of Yasuda et al. (2015), Sieljacks et al. (2016) and Bjørnsen et al. (2019) were all within the guidelines for BFR-RE in Table 1 in Patterson et al. (2019) with the exception of that of Sieljacks et al. (2016), which involved five sets instead of 2–4, but with only ~59 repetitions in total. Accordingly, we maintain that investigators, therapists and trainers should introduce BFR-RE protocols carefully and gradually progress them over time, to ensure that protective adaptations (i.e., a repeated bout effect) can take place in order to minimize the risk of excessive muscle stress and damage (Clark and Manini, 2017; Wernbom et al., 2019). Practitioners are also urged to recognize early signs of complications with BFR exercise and regularly report serious adverse events to enhance its safety and efficacy (Clark and Manini, 2017). Finally, despite over two decades of research on the neuromuscular adaptations to BFR-RE, it is apparent that the understanding of the training-overtraining-muscle damage continuum in BFR-RE is still in its infancy. Further in-depth research into these areas is urgently needed.

## Author Contributions

All authors listed have made a substantial, direct and intellectual contribution to the work, and approved it for publication.

### Conflict of Interest

The authors declare that the research was conducted in the absence of any commercial or financial relationships that could be construed as a potential conflict of interest.
